# A Delphi Study to Develop a Core Outcome Set for Capturing and Measuring Nurse Well‐Being

**DOI:** 10.1155/jonm/3582485

**Published:** 2026-08-31

**Authors:** Naomi Klepacz, David S. Baldwin, Gemma Simons

**Affiliations:** ^1^ Department of Health & Community Sciences, University of Exeter Medical School, Exeter, UK, exeter.ac.uk; ^2^ National Institute for Health Research (NIHR) Applied Research Collaboration Wessex (ARC Wessex), Southampton, UK; ^3^ University Department of Psychiatry, Clinical and Experimental Sciences, Faculty of Medicine, University of Southampton, Southampton, UK, southampton.ac.uk; ^4^ Hampshire and Isle of Wight Healthcare NHS Foundation Trust, Southampton, UK; ^5^ University Department of Psychiatry and Mental Health, University of Cape Town, Cape Town, South Africa, uct.ac.za; ^6^ Salisbury NHS Foundation Trust, Salisbury, Wiltshire, UK, salisbury.nhs.uk

**Keywords:** Core Outcome Set, Delphi study, NHS, nurse well-being, nursing workforce

## Abstract

**Background:**

Poor nurse well‐being is a significant concern, adversely affecting patient care quality and satisfaction and contributing to poor job satisfaction, increased sickness absence and workforce retention issues. There are calls for evidence‐based policies and interventions to address poor nurse well‐being, but no consensus exists on which outcomes should be captured to assess nurse well‐being. We used a salutogenic and consensus approach to develop a Core Outcome Set (COS) for nurse well‐being.

**Methods:**

A Delphi methodology was employed. Participants were recruited from two stakeholder groups: (1) nurse well‐being professionals, identified through relevant publications, conference/meeting attendance lists and peer recommendations, and (2) registered nurses working in the UK National Health Service (NHS), recruited via social media, professional nursing bodies and practitioner networks. The stakeholder panel completed two rounds of an online Delphi survey, rating 43 previously identified well‐being outcomes on a nine‐point Likert scale, from ‘not important’ to ‘critical.’ Consensus was defined as ≥ 75% of stakeholders agreeing a well‐being outcome was critical for inclusion in the COS.

**Results:**

Fifty‐four stakeholders completed the first Delphi round, and 45 participated in both rounds. Thirteen well‐being outcomes met the *a priori* threshold for inclusion in the COS: general well‐being, health, sleep, positive relationships, personal safety, psychological need satisfaction, psychological safety, job satisfaction, morale, life–work balance, compassion satisfaction, satisfaction with patient care and good nursing practice. The final COS was agreed by the stakeholder panel, without amendments.

**Conclusion:**

This study establishes a COS defining the nurse well‐being outcomes that should be captured when assessing nurse well‐being. Appropriate existing measurement instruments can be selected to capture these outcomes; future research is needed to identify and evaluate the most appropriate instruments and assess the feasibility of routinely capturing all 13 outcomes.

**Implications for Nursing Management:**

The COS provides nurse managers and workforce leaders with an agreed set of outcomes to support consistent nurse well‐being measurement, benchmarking and workforce monitoring. Its use can support evaluation of well‐being interventions, evidence‐informed decision‐making and the development of strategies and workplaces that enable nurses to thrive.

## 1. Introduction

Nurse well‐being is an important indicator of the state of the nursing workforce. Poor nurse well‐being impacts patient satisfaction and care quality, sickness absence and job satisfaction and leads to staff leaving the workforce [1–6]. Well‐being can be considered a continuum ranging from poor on one end to happiness, thriving and flourishing on the other [7]. However, most studies with nurses have focused on burnout (e.g., [8–10]) and psychiatric morbidity (e.g., [11, 12]), so little is known about positive (‘salutogenic’) indicators of well‐being in this profession [7]. International reviews continue to report moderate to high levels of burnout among nurses across a range of healthcare settings, and large‐scale workforce studies have linked burnout and psychological distress to intentions to leave the profession and wider workforce sustainability concerns [9, 10, 13]. However, such research predominantly focuses on occupational distress and pathologies, providing less insight into the positive factors that enable nurses to flourish and thrive.

Nurses constitute the largest group of clinical staff in the National Health Service (NHS), accounting for approximately 50% of the workforce [14]. Despite their deep commitment to providing high‐quality patient care, many nurses experience poor well‐being, stress and burnout [7, 15]. Physical and mental ill‐health, burnout and exhaustion currently follow retirement as the top reason nurses leave the profession [16]. A recent NHS staff survey reports that 42% of nurses found their work emotionally exhausting, 46% experienced work‐related stress and 58% came to work despite not feeling well enough to perform their duties (so‐called ‘presenteeism’) [17]. While nurses strive to prevent their own suboptimal well‐being from adversely affecting patient care [7], employers must recognise the direct link between nurse well‐being and patient safety and satisfaction [18]. Ensuring nurse well‐being is not only good for nurses themselves but is essential for the health and safety of patients and key to nurse retention.

Nurse well‐being is more than the absence of work‐related stress, injury or disease; it is achieving good physical and mental health amongst the nursing workforce [19]. Nurse leaders have a professional responsibility to create healthy working environments that promote and sustain well‐being. Managers, therefore, need a greater understanding of how nursing and the workplace impact nurse well‐being and how to engage with staff who need support [19]. Recent conceptual analyses have further described nurse well‐being as a multidimensional construct encompassing individual, organisational and community dimensions, with implications for leadership, workplace culture and policy [20]. Related nursing literature has similarly emphasised the dynamic and context‐dependent nature of well‐being‐related constructs such as resilience, recognising the importance of organisational and social factors rather than framing well‐being solely as an individual characteristic [21]. Recent (postpandemic) evidence indicates that resilience remains an important determinant of workforce sustainability, with low levels reported among nurses during and after COVID‐19 and resilience influenced by a range of individual, organisational and workplace factors [22, 23]. More broadly, international reviews have highlighted the enduring importance of nurse well‐being following the pandemic and emphasised the role of organisational and leadership support in sustaining a healthy nursing workforce [23].

Effective decisions and strategies to improve nurse well‐being must be grounded in reliable data, ensuring a robust evidence base [24]. Consistent and meaningful measurement is necessary to enable comparison across studies and synthesis of evidence to inform policy, practice and decision‐making. A sharper focus on the drivers of positive nurse well‐being is necessary to inform the development of policies, strategies and interventions that will enable the nursing profession to flourish and thrive. Recent UK policy initiatives [25] have emphasised the importance of creating organisational and cultural conditions that enable nurses and midwives to flourish at work. However, there remains no consensus on which well‐being outcomes should be captured and measured to support evaluation and evidence synthesis. Well‐being is a complex construct that includes measures and manifestations that have not yet been tested empirically among nurses [7]. It seems probable that no single measure can provide a complete picture of nurse well‐being.

A Core Outcome Set (COS) offers an agreed minimum for what should be captured, measured and reported [26]. Our study takes a salutogenic [27] and consensus approach to developing a COS defining the outcomes of nurse well‐being that should be captured and measured for nurses working in the NHS. Rather than conceptualising well‐being solely through the absence of pathology, a salutogenic approach focuses on the positive aspects of human experience and well‐being [27]. It is anticipated that the consistent capture, measurement and reporting of these outcomes will facilitate comparison by enhancing the ability to aggregate and analyse nurse well‐being data, which is necessary to support policy, strategy and intervention development. This work builds on our previous study to develop a COS for capturing and measuring the well‐being of doctors working in the NHS [28], so we have the additional objective of identifying potential convergence between the consensus outcomes for doctor and nurse well‐being.

## 2. Methods

### 2.1. Design

The study protocol was developed following Core Outcome Measures in Effectiveness Trials (COMET) criteria [29] and COS–STAndards for Development recommendations (COS‐STAD) [30]. It has similarities to our previous study on doctor well‐being [28]. The study was prospectively registered with the COMET Initiative [26] (Registration: 2433), and the findings are reported according to the COS–Standards for Reporting (COS‐STAR) guidance [31].

### 2.2. Stakeholder Recruitment

A purposive sampling strategy was adopted to recruit a participant panel from two stakeholder groups: (i) academics, policymakers, governance, occupational health professionals and support services staff, known here collectively as nurse well‐being experts, and (ii) registered nurses working in the NHS, considered experts by experience. Some overlap between the groups was anticipated, so participants were asked to self‐assign to a stakeholder group based on their primary job role. The inclusion criterion for the nurse well‐being experts group was as follows: individuals who have been or are involved in the concept, design, organisation, delivery, teaching, audit, governance, policy, guidance, research or well‐being of health and care professionals. We identified nurse well‐being experts from relevant healthcare workforce well‐being conferences, publications and special interest groups by searching previous conference proceedings, published guidelines and the well‐being literature. We further identified these stakeholders through recommendations from others. Potential participants were emailed a study invitation. All registered nurses working in the NHS were eligible to participate; an invitation was disseminated through our research, clinical academic and practitioner networks, social media, nursing professional bodies and nursing trade unions. Invitations included links to the participant information sheet, a brief video outlining the study and the online Delphi survey. Participants were required to complete a consent form, the first page of the online Delphi survey, before registering their details (name and email) and indicating which of the two stakeholder groups they identified with. Demographic data, including age, gender, geographical location, clinical speciality (for nurses), ethnicity and religion, were collected at registration to characterise the stakeholder panel and support interpretation of the findings. Each participant was assigned a study ID at registration, ensuring data were anonymous at the point of collection.

### 2.3. Outcomes and Domains

The starting point for this study was the list of 43 well‐being outcomes used previously in the development of the COS for doctor well‐being [28]. Using this set of outcomes allowed us to identify potential convergence between the consensus outcomes for doctor and nurse well‐being. However, the intention was not to assume direct transferability between professional groups. Rather, the Delphi process enabled nursing stakeholders to critically evaluate, refine and establish consensus regarding outcomes relevant to the nursing workforce. The 43 well‐being outcomes are categorised into five domains: (i) overall appraisal of well‐being, (ii) functional components of well‐being, (iii) activity and participation components of well‐being, (iv) work‐related well‐being and (v) health and care–specific well‐being [32]. The plain English descriptions of each outcome were reviewed for face validity, understanding and acceptability in a nursing context by our study advisory group (*n* = 6) and modified according to feedback (Table 1).

**TABLE 1:** Outcomes and their descriptions by domain.

Domain	Outcome	Description
Overall appraisal of well‐being	General well‐being	A state of positive feelings/affect/happiness and meeting full potential in the world (being the best person you can be in society). It can be measured subjectively and objectively using a salutogenic (positive) approach
	Meaning in life	Separate concept to well‐being, subjective sense of purpose, engagement with a philosophy of life or life goals and fulfilment
	Life satisfaction	Separate concept to well‐being, subjective appraisal of how much the person likes the life they lead; one of the indicators of quality of life.
	Quality of life	Separate concept to well‐being, subjective appraisal of an individual’s position in life in the context of the culture and value system in which they live and in relation to their goals, expectations, standards and concerns
	Wellness	Separate concept to well‐being, subjective or objective evaluation of the active pursuit of behaviours, choices and lifestyles that lead to a state of holistic health

Functional component of well‐being	Vitality	Relaxed possession of energy (physical, mental and emotional) and vigour; it is not actively strived for
	Optimism	Hopeful transcendence beyond (rising above) immediate circumstances
	Personality	Observable enduring characteristics/dispositions/tendencies to engage in certain patterns of behaviour
	Health	Subjective or objective evaluation of state of complete physical, mental and social well‐being, not merely the absence of disease or infirmity (the beneficial effects of green spaces, ability to relax, for example).
	Physiological function	Objective (snapshot) of body functions, i.e., electroencephalography (EEG), heart rate variability and electrodermal activity (temperature, sweating and cortisol levels).
	Cognitive function	Objective evaluation of domains such as, but not limited to, attention, memory and processing speed
	Self‐esteem	Self‐acceptance, self‐worth (pride) and sense of coherence (ability to predict events, belief in ability to manage them, that it is worth the effort, ability to be their true self and confidence in other achievements in nonwork‐related activities)
	Sleep	Subjective or objective evaluation of duration, quality and sense of feeling restored
	Financial security	Objective ability to pay for satisfactory accommodation, bills, care of dependants, ability to save for retirement, ability to cope with a sudden fall in income and ability to pay unexpected but necessary expenses

Activity and participation component of well‐being	Novelty	Subjective or objective growth through new experiences and learning (including posttraumatic growth)
	Positive relationships	Subjective or objective assessment of beneficial human connections (family and friends).
	Sexual well‐being	Subjective or objective assessment of sense of self and body, appreciating feelings of pleasure and desire, developing and maintaining mutually respectful gender equal relationships and safe and pleasurable sexual interactions
	Recreational activity	Subjective or objective evaluation of the ability to participate and participation in nonwork/leisure activities and the qualities of those chosen activities (example determinants are local investment and environment)
	Diet	Subjective or objective evaluation of the nutritional content, quantity and timing
	Physical activity	Subjective or objective assessment of the ability to participate in physical activity and the quality and quantity of physical exercise
	Engagement with preventative medicine	Subjective or objective assessment of participation in screening programmes they are eligible for and vaccines, accessing timely treatment

Work‐related well‐being	Financial reward satisfaction	Subjective or objective evaluation of ability to receive gratification from financial reward for effort (for example, satisfaction with pay and pension)
	Personal safety	Subjective or objective ability to go about work, and get to and from work, free from threat and safe from physical or psychological harm (infection, radiation, bullying, theft and assault)
	Psychological need satisfaction	Subjective or objective assessment of how autonomy (being in control of your life and work), belonging and competence needs have been supported by colleagues (inclusive and positive culture), managers (adequate workforce allows development) and supporting services (IT, administration, legal and occupational health)
	Psychological safety	Subjective or objective evaluation of the consequences of taking interpersonal risk at work (trust and information sharing)
	Job satisfaction	Subjective or objective evaluation of how much they like their choice of work profession, specialism and roles
	Morale	Subjective or objective evaluation of feelings about the future, ability of an individual, group or organisation to have and meet shared goals/values
	Engagement	Subjective or objective assessment of involvement and absorption with work and commitment to work
	Life–work balance	Subjective or objective quantity, quality and equality of time away from work and at work, the salience/clarity of the roles (the ability to work flexibly)
	Workability	Timely, objective assessment of having occupational competence and virtues, the health required for competence in an appropriate work environment by appropriate occupational health professionals
	Self‐care	Subjective or objective assessment of behaviours to look after own health and well‐being at work (taking breaks and time off work for sickness), accessing appropriate support services and adequate resources (estates, workforce and rapid‐access self‐referral services) to support this
	Professional development	Subjective or objective assessment of ability to participate and engage with learning and teaching knowledge and skills, and to progress
	Identification with work	Subjective or objective assessment of value and meaning assumed by the individual, or a group/team, at work (pride in work and professional identity)
	Resilience	Subjective or objective individual‐ or group‐level preservation of, or return to, previous function after exposure to trauma
	Emotional intelligence	Subjective or objective self‐awareness, self‐management, social awareness and relationship management
	Voice and influence	Subjective or objective assessment of ideas, concerns and expectations expressed informing policy and practice
	Confidence in leadership	Subjective or objective assessment of government and management competence, transparency and compassion, inclusivity, engagement and empowerment of those for whom they are responsible and accountable
	Recognition satisfaction	Subjective or objective evaluation of appreciation by colleagues, patients, public and government (civility)

Health and care specific well‐being	Compassion satisfaction	Subjective evaluation of ability to receive gratification from caregiving to patients, patients’ families and colleagues (satisfaction with nonfinancial rewards of the work)
	Altruism	Subjective or objective evaluation of selfless concern for the well‐being of others (patients and colleagues)
	Satisfaction with patient care	Subjective or objective assessment of quality of health and social care their patients receive from themselves and others (impacted by things such as staffing levels, competence, equipment, estates and funding available)
	Job plan/rota/rotation satisfaction	Subjective or objective evaluation of ability of role, responsibilities/rota/breaks to account for the quantity, types, of work (workload), the intensity, duration, of physical, mental and emotional demands and the rest/activities/resources needed to maintain it
	Good nursing practice	Subjective or objective assessment of ability to engage with complex or challenging patients/cases and advocate for them as indicated and in an evidence‐based way

### 2.4. Delphi Survey and Analysis

The Delphi technique aims to generate consensus by collecting opinions from stakeholder panel members and is widely used in developing COSs [33]. Using the online survey platform DelphiManager [34], we listed the 43 well‐being outcomes with plain English descriptions by domain. These were displayed in random order to participants. The Delphi survey was conducted over two rounds (Round 1 ran from 1 March 2023 to 24 March 2023, and Round 2 ran from 27 March to 30 April 2023). Adhering to the predefined Delphi survey guidelines [29], we asked participants to rate the importance of including each outcome in the COS using a 9‐point Likert scale. For analysis, ratings were grouped as follows: a rating of 1–3 on the Likert scale indicates the outcome is of ‘limited importance’ to include in the COS, a rating of 4–6 indicates the outcome is ‘important, but not critical’ to include, and a rating of 7–9 indicates that the outcome is ‘critical’ to include in a COS for the capture and measurement of nurses’ well‐being. These groupings were devised by the Grading of Recommendations Assessment, Development and Evaluation (GRADE) working group and have been used widely for Delphi methods [35]. Participants had the opportunity to provide a rationale for their ratings and were also given the option to indicate if they felt unable to score an outcome. At the end of each Delphi round, participants had the opportunity to suggest additional outcomes that they felt were not included among the 43 well‐being outcomes. Participants were advised that suggested outcomes should not be a symptom, sign or disease, nor a determinant of well‐being. The criterion for including suggested outcomes in the next Delphi round was that the published definition of the outcome differed significantly from the plain English descriptions of the existing outcomes. Participants who suggested an additional outcome were emailed by the research team, with the justification for including or excluding the outcome based on this criterion and were offered the opportunity to present further evidence or explanation.

In Round 2, the percentage of stakeholder panel members giving each rating for an outcome was fed back to participants. Summary scores were not provided by stakeholder group as the opinions of both were equally important to the final COS. Participants were also reminded of their own ratings from Round 1 and were given the opportunity to revise their ratings after reviewing the feedback. Three email reminders were sent to participants to encourage the completion of a round.

The criterion for outcomes to be included in the COS was defined *a priori* as ≥ 75% of all participants rating an outcome as ‘critical for inclusion’ (rating 7–9). This aligns with our previous study [28] and other similar COSs (e.g., [36–39]). The well‐being outcomes that met this threshold for inclusion in the COS were communicated to all stakeholder panel members via email, along with an invitation to provide further comments and/or endorse the final COS.

### 2.5. Ethical Approval

This study, which involved human participants, received approval from the University of Southampton Faculty Ethics Committee (ERGO 78343). Informed consent was obtained from all participants prior to their participation in this study.

## 3. Results

Study invitations were sent to 172 nurse well‐being experts, of whom 33 consented and registered to participate, yielding a response rate of 19.2%. In addition, 29 registered nurses agreed to participate, giving a total sample of 62 stakeholder panel participants. The mean age of participants was 48.1 years (range: 27–66 years); 48 participants (77.4%) self‐identified as female and 50 (80.7%) as White British. Four participants did not complete the survey (i.e., withdrew), and an additional four partially completed the survey. Partial responses were retained in the Round 1 analyses where participants had rated individual outcomes. In total, 54 participants (87.1%) rated all 43 well‐being outcomes (24 registered nurses and 30 nurse well‐being experts). Participants who rated some or all of the outcomes in Round 1 (*n* = 58) were invited to participate in Round 2, with 45 participants (18 registered nurses and 27 nurse well‐being experts) completing Round 2, giving a retention rate from Round 1 to Round 2 of 87%. All participants in Round 2 rated all outcomes.

In Round 1, six participants submitted eight suggestions for additional outcomes (Supporting Information 1). None of these suggestions met the criteria to be included for consideration in Round 2, either because the definition of an existing outcome already captured them or because they were pathologies (for example, two participants suggested burnout for inclusion). However, based on these recommendations and participant feedback, the definition of ‘Identification with work’ was amended to include ‘professional identity,’ and the definitions of ‘Self‐esteem’ and ‘Identification with work’ were amended to include ‘pride.’ None of the 43 outcomes was removed following Round 1. No additional outcomes were suggested in Round 2.

At the end of Round 2, 13 outcomes met the ≥ 75% threshold for inclusion in the COS for capturing and measuring nurse well‐being. These outcomes were general well‐being, health, sleep, positive relationships, personal safety, psychological need satisfaction, psychological safety, job satisfaction, morale, life–work balance, compassion satisfaction, satisfaction with patient care and good nursing practice (Table 2). The full Round 2 outcomes’ ratings are presented in Supporting Information 2. Outcomes were subsequently emailed to participants for further comment and review. Participants agreed to the final COS without further amendments.

**TABLE 2:** Final Core Outcome Set.

Domain	Outcome	% Participants rating outcomes as ‘critical’
Overall appraisal of well‐being	General well‐being^∗^	93.18

Functional component of well‐being	Health^∗^	84.44
	Sleep	88.89

Activity and participation component of well‐being	Positive relationships	86.67

Work‐related well‐being	Personal safety^∗^	80.00
	Psychological need satisfaction	91.11
	Psychological safety	88.89
	Job satisfaction^∗^	91.11
	Morale^∗^	75.56
	Life–work balance^∗^	80.00

Health and care specific well‐being	Compassion satisfaction	77.78
	Satisfaction with patient care	93.33
	Good nursing practice^∗^	77.78

^∗^Outcomes also included in the Core Outcome Set for capturing and measuring doctor well‐being [28]. ‘Good nursing practice’ in the nurse COS corresponds to ‘Good Clinical Practice’ in the doctor COS.

## 4. Discussion

This study developed the first COS specifically for the well‐being of nurses working in the NHS. The stakeholder panel of registered nurses and nurse well‐being experts reached a consensus on a minimum set of 13 outcomes that should routinely be captured and measured for nurse well‐being. We recommend that future data collection initiatives adopt this COS to ensure standardisation, enabling a consistent, comparable and comprehensive evidence base with the potential to support decision‐making for policy and practice. Previous research has focused on determinants and interventions of nurse well‐being rather than the outcomes that might demonstrate how these determinants or interventions influence this profession. By creating this COS and promoting its use, we seek to shift the current discourse towards an understanding of positive (salutogenic) components of nurse well‐being. This shift is critical for the development of effective well‐being policies, strategies and interventions that empower nurses to flourish in the workplace. Future research is now needed to identify and evaluate outcome measurement instruments.

A strength of our approach is that it provides outcomes for each of the five well‐being domains. Several agreed‐upon well‐being outcomes, such as morale, personal safety and job satisfaction, are already captured through, for example, the NHS staff survey [40] and the RCN Employment survey [41]. The inclusion of these outcomes within established workforce monitoring initiatives supports their practical relevance and suggests that they represent aspects of well‐being considered important to the nursing workforce. Other outcomes, such as ‘good nursing practice,’ will require the identification of outcome measurement instruments based on their descriptions in Table 1. Furthermore, the seven well‐being outcomes that comprise the COS for capturing and measuring doctor well‐being (general well‐being, health, personal safety, job satisfaction, morale, life–work balance and good clinical practice) [28] met the threshold for inclusion in the COS for nurses’ well‐being. This alignment suggests that factors considered relevant to doctors’ well‐being are similarly relevant to nurses. Indeed, previous research indicates that certain features of work‐related well‐being and mental ill‐health are common across all NHS staff groups [6]. However, the additional outcomes identified for the COS for nurse well‐being underscore important profession‐specific differences that must be considered when developing policies, strategies and interventions for nurses. While these examples are drawn from the UK context, many of the agreed outcomes reflect aspects of well‐being that may be relevant across healthcare systems, although further work is required to establish the applicability of the COS internationally.

The robust and replicable methodology used in this study followed the COS‐STAD guidelines [30] and built on our previous work developing a COS for capturing and measuring doctor well‐being [28]. The long list of outcomes presented to the stakeholder panel was evidence‐based [32], and our study advisory group ensured the relevance and validity of this list to nursing. The presentation of domains to the stakeholder panel participants was randomised using the DelphiManager platform [34] to avoid presentation bias. Furthermore, additional well‐being outcomes suggested by participants during the Delphi survey were already represented by existing well‐being outcomes, further supporting the comprehensiveness of the long list. This suggests that the final COS reflects prioritisation of well‐being outcomes considered most important by stakeholders, rather than the omission of well‐being components identified elsewhere in the literature. The suggestions to add burnout as a well‐being outcome reflect the current use of burnout and psychiatric morbidities as proxies for well‐being, further underscoring the need for this COS. While the lack of an internationally agreed‐upon operational definition of nurse well‐being may be seen as a limitation, we addressed this by utilising our published operational definition of well‐being [27] and applying a salutogenic, consensus‐based methodology. This approach enabled us to establish a panel‐agreed COS for well‐being outcomes relevant to nurses.

A further strength of this study was that it included registered nurses and nurse well‐being professionals in the stakeholder panel. However, we acknowledge that stakeholders outside the present panel might have differing views. The stakeholder panel was predominantly White British, and perspectives on nurse well‐being may differ across racial and ethnic groups; further work is needed to examine the relevance and acceptability of the COS within more racially and ethnically diverse nursing populations. The sample size was appropriate for a Delphi study [30], as was the response rate from nurse well‐being professionals to the invitation and the overall retention rate. Our focus on nurses working in the UK’s NHS means that stakeholders were invited accordingly. The recruitment method for registered nurse stakeholders was designed to reach all nurses working in the NHS; however, we are unaware of how many potential participants saw our invitation and elected not to participate. While this COS might be relevant to nurses working in other healthcare systems, both in the United Kingdom and beyond, additional investigation is required to ensure its broader applicability across different healthcare contexts.

We acknowledge that users of this COS may find it challenging to capture and measure all 13 outcomes that comprise this COS, and it should be noted that the feasibility of using this COS on every occasion nurse well‐being is measured has not yet been tested. While we suggest these outcomes as a minimum, users may wish to include other outcomes relevant to their research or capture and measure only those outcomes from their domain of interest; for example, the work‐related well‐being domain with its six well‐being outcomes or the subset of seven well‐being outcomes common to both doctors and nurses. The robust methodology we have applied in this study could be repeated to assess the relevance of these outcomes to other healthcare professions. This COS provides a framework to better understand positive components of well‐being in the nursing profession, and in line with COMET guidelines [29], our next step is to identify which outcome measurement instruments would be most appropriate and accessible for end users.

### 4.1. Implications for Nursing Management

The COS provides nurse managers and workforce leaders with an agreed set of outcomes to support a consistent approach to capturing nurse well‐being. Adoption of these outcomes across services could facilitate benchmarking and workforce monitoring, while enabling evaluation of whether well‐being interventions and workplace changes result in meaningful improvements for nurses. The resulting evidence can support leadership decision‐making, inform the development of nurse well‐being strategies and help identify where action is needed to create workplaces that enable nurses to thrive.

## 5. Conclusion

This study has identified a minimum set of well‐being outcomes that should be used when measuring NHS nurse well‐being. Implementing this COS will reduce heterogeneity in the outcomes captured, facilitating evidence synthesis and benchmarking to better understand the current state of nurse well‐being . Future efforts will focus on identifying and evaluating the most appropriate instruments for measuring these outcomes.

## Author Contributions

• Gemma Simons and David S. Baldwin devised the study and acquired funding.

• Naomi Klepacz designed the study; collected, analysed and interpreted the data; wrote the manuscript; and edited and approved the final manuscript.

• Gemma Simons and David S. Baldwin supervised the study’s design, data collection, analysis and interpretation and approved the final manuscript.

• Gemma Simons acts as guarantor.

## Funding

This study was funded by the National Institute for Health and Care Research (NIHR) Applied Research Collaboration Wessex (ARC Wessex) Mental Health Hub. For the purpose of open access, the author has applied a CC BY public copyright licence to any Author Accepted Manuscript arising from this submission.

## Disclosure

The views expressed in this publication are those of the authors and not necessarily those of the National Institute for Health and Care Research or the Department of Health and Social Care.

A preprint of this manuscript has been published on medRxiv[42].

This study was prospectively registered with the COMET Initiative https://www.comet-initiative.org (Registration no: 2433).

## Ethics Statement

This study involved human participants and was approved by the University of Southampton Faculty Ethics Committee (ERGO 78343). Participants gave informed consent before participating.

## Consent

Please see the Ethics Statement.

## Conflicts of Interest

The authors declare no conflicts of interest.

## Supporting Information

Additional supporting information can be found online in the Supporting Information section.

## Supporting information


**Supporting Information 1** Supporting Information 1 presents additional well‐being outcomes suggested by participants during Round 1 of the Delphi survey, along with the rationale for their exclusion from subsequent rounds.

**Supporting Information 2** Supporting Information 2 provides detailed Round 2 outcome ratings, including the percentage of participants rating each outcome as being of limited, important or critical importance.

## Data Availability

All data relevant to the study are included in the article or uploaded as Supporting Information.
