# Supplementary material for: A Delphi Study to Develop a Core Outcome Set for Capturing and Measuring Nurse Well‐Being
Source: J Nurs Manag. 2026 Aug 31;2026:3582485. doi: 10.1155/jonm/3582485 (PMC13530399; doi:10.1155/jonm/3582485)
Supplement: Supplementary file 1 [file JONM-2026-3582485-s001.docx]

**Supplementary Materials 1**. Additional outcomes suggested by stakeholders in Round 1 with rationale for not including as unique outcomes in Round 2.

|  | **Suggested New Outcomes** | **Rationale for not including as a unique outcome in Round 2** |
| --- | --- | --- |
| 1 | Sharing concerns with others | **Not novel, captured by existing outcomes.** This is captured by the outcome ‘Psychological safety’. |
| 2 | Supportive colleague interaction/relationships | **Not novel, captured by existing outcomes.** This is captured by the outcome ‘Psychological safety’ |
| 3 | Burnout | **Diagnosis or pathology.** We chose not to include mental health diagnoses as wellbeing outcomes, as these are separate concepts. |
| 4 | Belonging | **Not novel, captured by existing outcomes.** This is captured by the outcome ‘Psychological needs satisfaction’ |
| 5 | Human flourishing | **Not novel, captured by existing outcomes.** This is captured by the outcomes ‘Vitality’ and ‘Novelty’ |
| 6 | Professional identity | **Not novel, captured by existing outcomes.** This is captured by the outcome ‘Identification with work’. |
| 7 | Pride (in being part of the organisation for which the nurse works) | **Not novel, captured by existing outcomes.** This is captured by the outcomes ‘Self-esteems’ and ‘Identification with work’. |
| 8 | Burnout | **Diagnosis or pathology.** We chose not to include mental health diagnoses as wellbeing outcomes, as these are separate concepts. |
